# Glia and Muscle Sculpt Neuromuscular Arbors by Engulfing Destabilized Synaptic Boutons and Shed Presynaptic Debris

**DOI:** 10.1371/journal.pbio.1000184

**Published:** 2009-08-25

**Authors:** Yuly Fuentes-Medel, Mary A. Logan, James Ashley, Bulent Ataman, Vivian Budnik, Marc R. Freeman

**Affiliations:** Department of Neurobiology, University of Massachusetts Medical School, Worcester, Massachusetts, United States of America; Stanford University School of Medicine, United States of America

## Abstract

Synapse remodeling is an extremely dynamic process, often regulated by neural activity. Here we show during activity-dependent synaptic growth at the *Drosophila* NMJ many immature synaptic boutons fail to form stable postsynaptic contacts, are selectively shed from the parent arbor, and degenerate or disappear from the neuromuscular junction (NMJ). Surprisingly, we also observe the widespread appearance of presynaptically derived “debris” during normal synaptic growth. The shedding of both immature boutons and presynaptic debris is enhanced by high-frequency stimulation of motorneurons, indicating that their formation is modulated by neural activity. Interestingly, we find that glia dynamically invade the NMJ and, working together with muscle cells, phagocytose shed presynaptic material. Suppressing engulfment activity in glia or muscle by disrupting the Draper/Ced-6 pathway results in a dramatic accumulation of presynaptic debris, and synaptic growth in turn is severely compromised. Thus actively growing NMJ arbors appear to constitutively generate an excessive number of immature boutons, eliminate those that are not stabilized through a shedding process, and normal synaptic expansion requires the continuous clearance of this material by both glia and muscle cells.

## Abstract 2

As synapses grow at the *Drosophila* neuromuscular junction, they shed membrane material in an activity-dependent manner. Glia and postsynaptic muscle cells are required to engulf this debris to ensure new synaptic growth.

## Author Summary

The synapse is the fundamental unit of communication between neurons and their target cells. As the nervous system matures, synapses often need to be added, removed, or otherwise remodeled to accommodate the changing needs of the circuit. Such changes are often regulated by the activity of the circuit and are thought to entail the extension or retraction of cellular processes to form or break synaptic connections. We have explored the precise nature of new synapse formation during development of the *Drosophila* larval neuromuscular junction (NMJ). We find that growing synapses are actually quite wasteful and shed significant amounts of presynaptic membranes and a subset of immature (nonfunctional) synapses. The shedding of this presynaptic material is enhanced by stimulating the activity of the neuron, suggesting that its formation is dependent upon NMJ activity. Surprisingly, we find presynaptic membranes are efficiently removed from the NMJ by two surrounding cell types: glia cells (a neuronal ‘support cell’), which invade the NMJ, and the postsynaptic muscle cell itself. Blocking the ability of these cells to ingest shed presynaptic membranes dramatically reduces new synapse growth, suggesting that the shed presynaptic material is inhibitory to new synapse addition. Therefore, our data demonstrate that actively growing synapses constantly shed membrane material, that glia and muscles work to rapidly clear this from the NMJ, and that the combined efforts of glia and muscles are critical for the proper addition of new synapses to neural circuits.

## Introduction

The wiring of the nervous system, from initial axon sprouting to the formation of specific synaptic connections, represents one of the most dramatic and precise examples of directed cellular outgrowth. Developing axons navigate sometimes tortuous routes as they seek out the appropriate target cells. Once in their target area, interactions between axons and their potential targets are extremely dynamic, attempts are made to identify appropriate postsynaptic partners, and initial synaptic contacts are established [1],[2],[and reviewed in 3]. A next critical step in the formation of functional neural circuits is the remodeling of initial patterns of connectivity. To facilitate the elaboration and refinement of developing neural circuits synaptic partners often remain highly responsive to their environment and add or eliminate synaptic connections [4],[5], frequently in an activity-dependent fashion, presumably to fine-tune connectivity to specific activity patterns.

After the axons have found their partners, two distinct mechanisms can drive the developmental reorganization of synaptic connectivity: intercellular competition between cells for common targets (reviewed in [4],[5]), and the addition/elimination of synapses within a single arbor in response to the physiological demands of the signaling unit [6]–[8]. The former mechanism dictates the circuit “wiring diagram” by defining precisely which subsets of cells will communicate through synaptic contacts; while the latter, in contrast, modulates the strength of connectivity between specific pre- and postsynaptic cells after circuits are assembled.

Early in nervous system development an excessive number of axonal projections and synaptic connections are initially established. What then ensues is cell–cell competition between neurons innervating the same target for limiting target-derived cues or sites of innervation during synaptogenesis. Appropriate synaptic contacts are then strengthened and exuberant processes are destabilized and eliminated through activity-dependent mechanisms [5],[9]. For example, at the mammalian neuromuscular junction (NMJ) muscles are initially innervated by more than one motor input. However, through a process of intercellular competition for motor endplates, all but one motor input are eliminated, with the “losers” retracting wholesale from the motor endplate [2]. Likewise, at the retinotectal projection in frogs, retinal axons initially establish a rough topographic map with substantial overlap between branches. However, these local synaptic terminals ultimately compete for target space and through activity-dependent modulation of synapse stabilization the spatial map of synaptic inputs is ultimately refined to a highly selective subset of inputs [10].

In the intercellular competition model the elimination of exuberant inputs (the “losers”) can entail large-scale elimination of axon branches, and perhaps smaller scale pruning of individual synaptic contacts. During axon and synaptic pruning in mammals and *Drosophila* entire axon branches are destabilized, degenerate, and are then cleared from the central nervous system by engulfing cell types (reviewed in [5]). Similarly, recent work has shown that excessive motorneuron inputs at the mammalian NMJ also become destabilized, detach from the motor endplate, and undergo axosome shedding. In this process local Schwann cells processively engulf motorneuron terminals in a distal to proximal direction and constitute the force that drives retraction bulbs toward the parent arbor during input elimination [11]. Ultimately, this mechanism results in a reduction of the total number of cells supplying synaptic input to the target cell.

In the second and mechanistically distinct mode of synapse remodeling, individual synaptic contacts are added or removed from a single arbor to strengthen or weaken synaptic input to the target cell. Such changes are generally elicited by changes in the target size or neural activity. For example, *Drosophila* motorneurons have established synaptic contacts with specific embryonic muscle cells by the end of embryogenesis [12]. At subsequent larval stages individual arbors, along with the target muscle itself, grow in size ∼100-fold [7],[8]. This coordinate increase in muscle size and synaptic contacts at motorneuron terminals serves to increase synaptic input from the motorneuron as needed to drive activation of the expanding muscle fiber. Similar mechanisms appear in place to modulate the balance of neural input versus target cell size in mammals: at the mammalian adult bulbocavernous muscle, testosterone manipulation lead to increases or decreases in muscle size, and these changes were accompanied by respective expansion or shrinkage of the postsynaptic region of the NMJ, respectively [6].

Here we explore the in vivo dynamics of synaptic expansion in motorneuron arbors at the *Drosophila* NMJ. We show in live preparations that the addition of new synapses during normal synaptic growth entails a large amount of shedding of presynaptic membranes in the form of small debris and a subpopulation of undifferentiated synaptic boutons (ghost boutons) that failed to mature. This process is distinct from intercellular competition, as none of the motorneuron terminals are eliminated. Rather, this mechanism appears to regulate the final size of the terminal arbor. We find that the formation of presynaptic debris (this report) and ghost boutons [13] are modulated by neural activity, as acute stimulation of motor inputs leads to increased appearance of these structures. Intriguingly, presynaptic debris and the subpopulation of ghost boutons that become detached from the parent arbor appear to be actively cleared from the NMJ as they disappear over developmental time. We show that glia dynamically invade the NMJ and phagocytose presynaptically shed debris, and that ghost boutons are engulfed or degraded primarily by muscle cells. Loss of phagocytic function in glia or muscle cells through manipulating the Draper signaling pathway (a key engulfment signaling pathway) results in an accumulation of presynaptic debris or ghost boutons at the NMJ and a severe reduction in NMJ expansion, indicating that continuous clearance of shed presynaptic debris and/or ghost boutons is essential for normal synaptic growth. Thus glia and muscles work together to sculpt connectivity at developing NMJ arbors, clearing multiple types of shed presynaptic structures that are inhibitory to the formation of new synaptic boutons.

## Results

### The Larval NMJ Sheds Presynaptic Membranes

**Figure 1:**
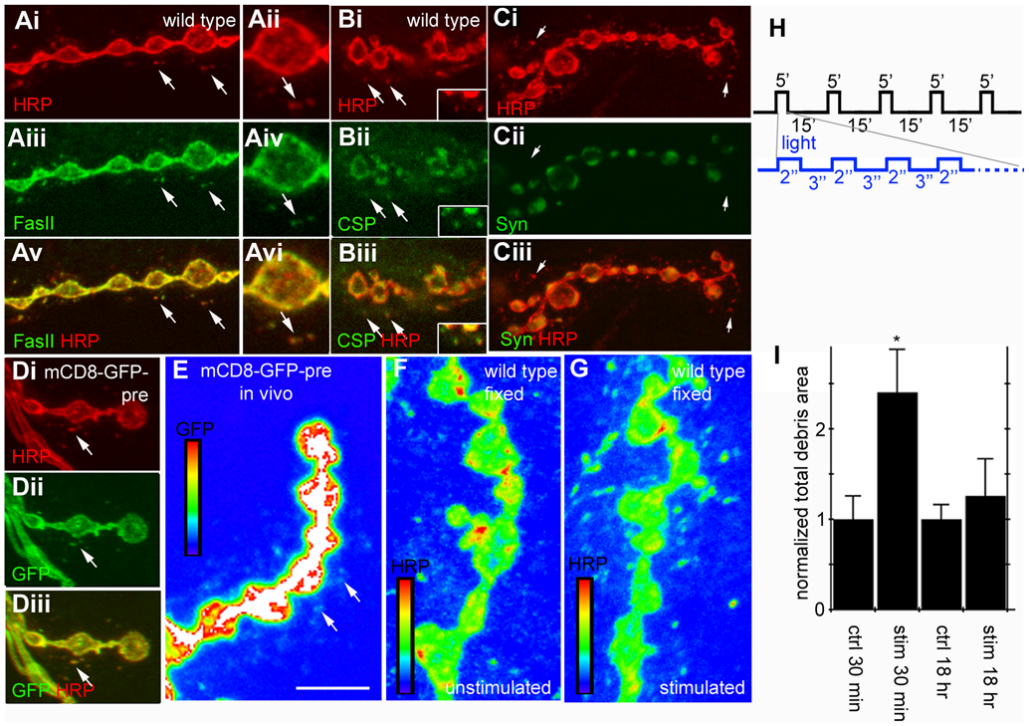
Motor axons at the NMJ constitutively shed presynaptic debris in an activity-dependent manner. (A–D) Third instar Drosophila larvae were fixed and stained with various markers to visualize the morphology of glutamatergic NMJ branches at muscles 6 and 7. All motor neurons labeled with α-HRP (red) and small HRP^+^ puncta were observed adjacent to many NMJ arbors (arrows, Ai–Diii). These puncta colocalized with the motor neuron marker FasII (green, Ai–vi) and the synaptic vesicle marker CSP (green, Bi–iii). Presynaptically derived HRP^+^ debris does not stain for Syn (green in Ci–iii), a marker for reserve pools of synaptic vesicles. (D, E) UAS-mCD8-GFP was driven in motor neurons with the OK6-Gal4 driver. All HRP^+^ puncta were also GFP^+^ in fixed samples (arrows, Di–iii), indicating that the HRP^+^ puncta are presynaptically derived. Presynaptically derived GFP+ debris was also observed in live, intact animals by imaging through the cuticle. (F–I) Unstimulated NMJs display very little or no HRP^+^ debris surrounding NMJ arbors (F). Spaced light stimulation of larvae expressing presynaptic channelrhodopsin-2 led to a dramatic increase in the formation of HRP^+^ presynaptic debris surrounding the NMJ 30 min after stimulation ended (G, H). (H) Light stimulation paradigm where 5 pulses of 5 minute stimulations (divided into repeating 2 seconds on 3 seconds off) are spaced with 15 minutes of rest. (I) Quantification of normalized total area of HRP^+^ debris. Calibration scale is 10 µm for (A–D) and 6 µm for (E–F). *n* = 18,12, 6, 6, respectively for (I).

In insects, α-HRP antibodies cross-react with neuron-specific membrane antigens [14] likely by binding to carbohydrate moieties present in a number of neuronal membrane proteins, including the cell adhesion molecules Fasciclin (Fas) I and II [15]. Consistently, at the *Drosophila* larval NMJ α-HRP antibodies labeled the entire presynaptic arbor (Figure 1Ai). However, we also noticed the presence of HRP-immunoreactive puncta at the postsynaptic junctional region, beyond the presynaptic membrane (Figures 1Ai, 1Aii, arrows). These puncta also labeled with antibodies to FasII and did not appear to be connected to the presynaptic arbor (Figures 1Aiii, Aiv). We wondered whether the HRP and FasII-positive postsynaptic staining might correspond to postsynaptic muscle structures, or whether the puncta might be derived from the presynaptic arbor. To distinguish between these possibilities, we expressed a membrane tethered green fluorescent protein (GFP; UAS-mCD8-GFP) in motorneurons using the motoneuron-specific Gal4 driver OK6-Gal4 [16]. We found that the postsynaptic HRP puncta were exactly colocalized with the presynaptically derived GFP signal (Figure 1D, arrow), suggesting that the HRP puncta are likely membrane fragments derived from presynaptic boutons. The presynaptically derived mCD8-GFP puncta were also observed by imaging through the cuticle of intact (undissected) larvae using a spinning disk confocal microscope, indicating that they are naturally occurring and not an artifact of the dissection or sample preparation (Figure 1E, arrows).

The nature of the presynaptically derived puncta was examined using a number of synaptic markers. Cysteine string protein (CSP) and Synapsin (Syn) are presynaptic vesicle proteins that associate with the readily releasable and the reserve pool of synaptic vesicles respectively [17],[18]. We found that the postsynaptic HRP puncta colocalized with CSP (Figure 1B, arrows and inset), but not with Syn immunoreactivity (Figure 1C). The presence of CSP in the HRP puncta further validates the idea that these puncta are presynaptic in origin. Labeling with antibodies against the active zone marker Bruchpilot (Brp) did not reveal immunoreactivity at the postsynaptic HRP-positive puncta (unpublished data). Together these results suggest that during NMJ development the motorneuron sheds membrane fragments (here referred to as presynaptic debris). Based on the presence of CSP but not Syn, the absence of Brp and the presence of FasII, we propose that presynaptic debris might arise from the perisynaptic bouton region.

Studies in many systems have suggested that the state of a mature synapse is the result of a dynamic equilibrium between growth and retraction [19]. Therefore, to determine what conditions lead to the shedding of presynaptic debris, we attempted to perturb this equilibrium by inducing activity-dependent synaptic growth [13]. Previous studies at the larval NMJ show that an acute increase in activity induces a de novo formation of new synaptic boutons. In particular, spaced cycles of stimulation, consisting of either K^+^-induced depolarization, high frequency nerve stimulation, or light gating of neuronally expressed channelrhodopsin-2 (ChR2), induce rapid structural changes at the NMJ. These changes include an increase in the number and length of dynamic presynaptic filopodia (synaptopods) and the number of undifferentiated boutons (ghost boutons) containing synaptic vesicles but lacking active zones and postsynaptic proteins [13]. Imaging of intact larvae also showed that synaptopods and ghost boutons were naturally occurring structures observed even in unstimulated preparations albeit at low frequency [13].

In our experiments we expressed ChR2 in motorneurons using OK6-Gal4 and stimulated the motorneurons of intact larvae with 5 cycles of spaced light stimulation as previously described [13]. Body wall muscles were then dissected either 30 min or 18 h after the stimulation was complete and labeled with anti-HRP. As a control, we used unstimulated larvae expressing ChR2 in motorneurons but not subjected to the light pulses. Notably, we found that the total area occupied by particles of presynaptic debris around the NMJ was significantly increased 30 min after the end of spaced stimulation (Figure 1F–1I), indicating that acute stimulation of neural activity resulted in an increase in presynaptic debris at the NMJ. Allowing NMJs to recover for 18 h after stimulation resulted in debris returning to wild-type levels (Figure 1I), suggesting the presence of an active mechanism to eliminate presynaptic debris from the NMJ. We conclude that presynaptic debris are normally present at the NMJ and conditions that lead to synaptic growth result in a transient increase in the amount of presynaptic debris, thus shedding of debris is associated with NMJ growth.

**Figure 2:**
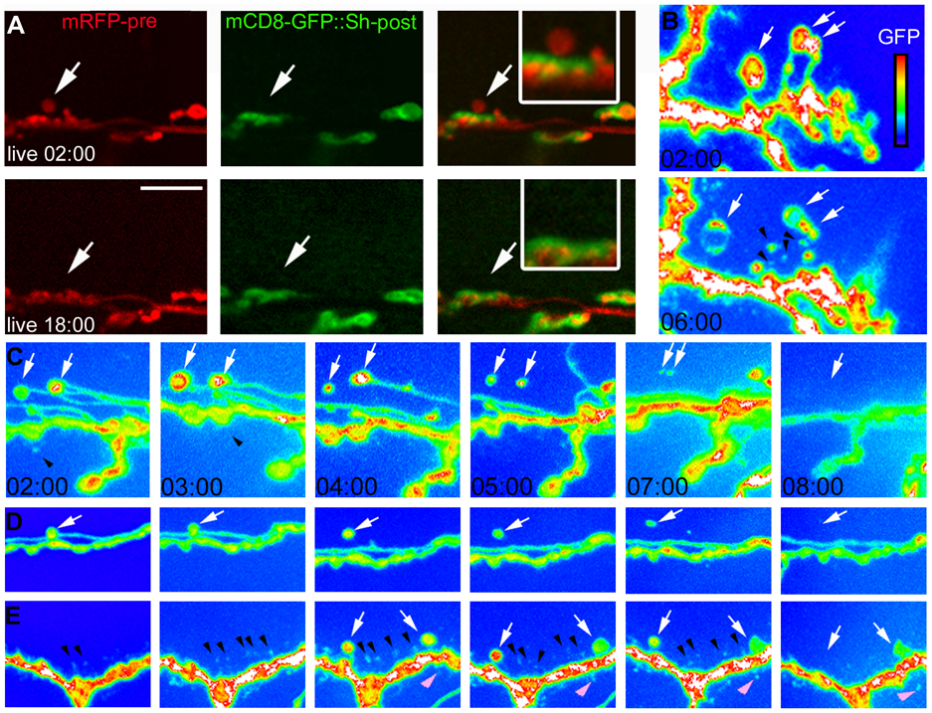
NMJs shed ghost boutons that stabilize or disappear. (A) Example of live imaging of an NMJ through the cuticle of an intact larvae expressing channelrhodopsin-2 and mRFP (red) in motoneurons, and a synaptically targeted mCD8-Shaker-GFP protein (green) in postsynaptic muscles. Motor neurons were stimulated with a spaced blue light paradigm (as in Figure 1) and NMJs were imaged at indicated times. Stimulation led to the formation of a ghost bouton (arrow) that lacked postsynaptic mCD8-Shaker-GFP. 18 h later, the ghost bouton was eliminated. (B) Live, intact larvae expressing channelrhodopsin-2 and mCD8-GFP in motor neurons were imaged immediately and 4 h after spaced light stimulation. White arrows point to ghost boutons observed before and after stimulation. Black arrowheads point to presynaptic debris that formed after stimulation. (C–E) Live, intact larvae expressing channelrhodopsin-2 and mCD8-GFP in motor neurons were imaged immediately and at 1-h intervals after spaced light stimulation. In some instances, detached ghost boutons simply became smaller and disappeared leaving debris (C and D, arrows), while detached ghost boutons sometimes simply became smaller and disappeared without leaving any obvious debris (E, white arrows) Presynaptic debris at NMJ regions devoid of ghost boutons would also appear and then disappear following stimulation (E, black and pink arrowheads). Calibration scale is 17 µm for (A, and C–E), 12 µm for (B), and 9 µm for (A, inset). Times correspond to hours from beginning of experiment when preparations were first imaged.

We also conducted time-lapse imaging of identified NMJs from live intact larvae expressing ChR2 in motorneurons using C380-Gal4 [20]. These larvae also contained fluorescent markers that allowed us to simultaneously image the pre- and the postsynaptic compartment. In particular, these larvae expressed UAS-mRFP in motorneurons to visualize the presynaptic NMJ arbor and mCD8-GFP::Sh in muscles using the myosin heavy chain (MHC) promoter [21] to visualize the postsynaptic NMJ region. In the MHC-mCD8-GFP::Sh transgene, the GFP is fused to the last ∼150 C-terminal amino acids of the Shaker K^+^ channel isoform containing a Discs-Large (DLG) PDZ binding site, and thus it is targeted to the postsynaptic region allowing its visualization in vivo [21]. These larvae were subjected to spaced stimulation with light as above, and the same NMJ imaged for 5–15 min at different intervals. Between imaging intervals larvae were returned to the food. As previously reported [13], we found that ghost boutons were present and some of these became stabilized and recruited postsynaptic label. However, we also observed that many of these ghost boutons did not recruit postsynaptic label and disappeared over time (Figure 2A, arrow and inset in right panel).

The presence of presynaptic debris in normal animals, the enhancement of presynaptic debris deposition upon spaced stimulation, and the elimination of some of the newly generated ghost boutons after spaced stimulation suggest that NMJ development involves the continuous shedding of certain presynaptic membrane compartments. Furthermore, the lack of accumulation of these components over developmental time, suggest that they may be actively removed from the NMJ.

To determine if presynaptic debris might originate from the breakdown of ghost boutons that failed to become stabilized and disappeared, we followed the fate of ghost boutons that became detached from the presynaptic arbor and presynaptic debris. In these experiments, identified NMJs from larvae expressing ChR2 and mCD8-GFP in motorneurons were repeatedly imaged through the cuticle as above following spaced stimulation. We found that on several occasions, as ghost boutons detached, debris appeared in the position of the ghost bouton stalk and around the ghost bouton, suggesting that ghost boutons can degenerate directly into presynaptic debris (e.g., Figure 2B and 2C; ghost boutons are marked by white arrows and debris by black arrowheads). In some samples we were able to directly image the disintegration of ghost boutons into smaller fragments (Video S1). However, in other cases, stalks simply disappeared without leaving debris, and detached ghost boutons became smaller and vanished from the NMJ without leaving any obvious debris (Figure 2D and 2E, white arrows). Interestingly, not all presynaptic debris appeared to derive from ghost boutons and their stalks—we also observed the appearance and disappearance of presynaptic debris at NMJ regions devoid of ghost boutons (Figure 2E, black and pink arrowheads), suggesting that presynaptic debris can be generated independently from ghost boutons. In summary, presynaptic debris can apparently arise directly from the breakdown of ghost boutons, or, alternatively may be derived directly from the presynaptic arbor without participation of ghost boutons.

### Local Engulfing Cells Clear Shed Presynaptic Material from the NMJ

**Figure 3:**
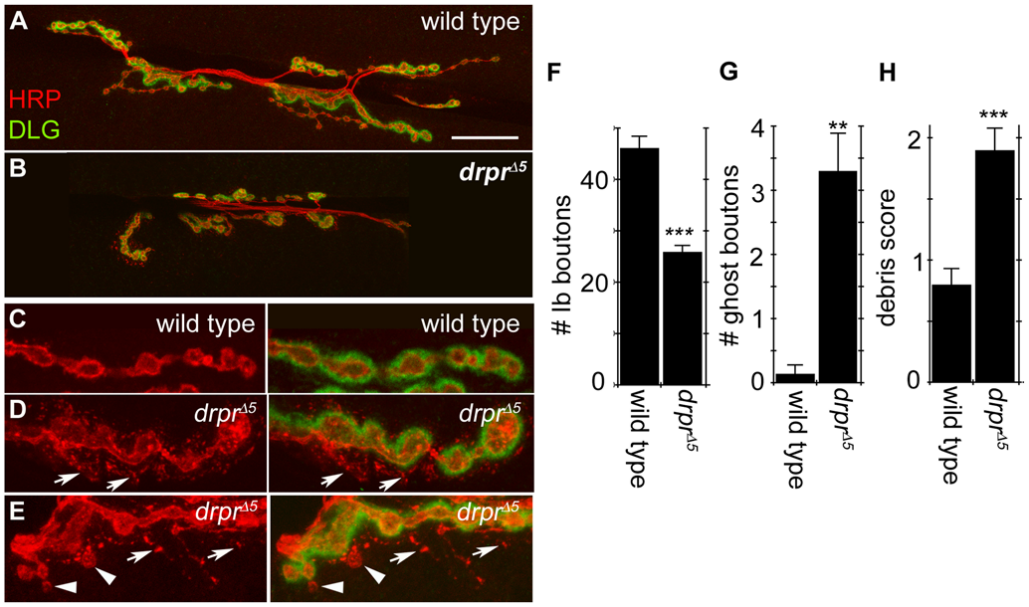
*draper* mutant NMJs exhibit reduced synaptic growth and accumulate pruned ghost boutons and presynaptic debris. (A) A wild-type third instar NMJ at muscles 6/7 visualized with α-HRP (red) and the postsynaptic marker DLG (green). (B) *draper* ^Δ*5*^ mutants have disrupted NMJ morphology and a significant reduction in the number of type Ib boutons compared to wild type. (C) The NMJ in wild-type animals normally has very little presynaptic debris and ghost boutons are only rarely observed. (D, E) The NMJ in *draper* ^Δ*5*^ mutants accumulates large amounts of shed presynaptic debris (arrows) and many ghost boutons (arrowheads). (F–H) Quantification of the number of (F) type Ib boutons, (G) ghost boutons, and (H) presynaptic debris at muscles 6/7. ***, *p*<0.001; **, p≤0.01; *, *p*≤0.05. Calibration scale is 25 µm for (A and B), 8 µm for (C–E). (*n* = 9 for both wild type and *draper* ^Δ*5*^).

The very low levels of presynaptic debris and ghost boutons observed here in unstimulated larvae and the removal of the extra debris formed upon stimulation, suggested that as NMJs develop, presynaptic membrane debris and disconnected ghost boutons are actively cleared from the NMJ. Signal transduction mechanisms mediating the engulfment of neuronal debris are beginning to be elucidated [22]. Most prominent, the engulfment receptor Draper (Drpr; Ced-1 in *Caenorhabditis elegans*) is involved in the engulfment of neuronal cell corpses during programmed cell death, the pruning of mushroom body neuron arbors during fly metamorphosis, and in the phagocytosis of injured axons in the fly olfactory system [23]–[26]. We therefore used *draper* mutants as a tool to block the activity of local engulfing cell types and assayed the effects of loss of Draper function on clearance of shed presynaptic debris and disconnected ghost boutons from the larval NMJ. Strikingly, we found that *draper* mutant NMJs were highly abnormal, with the presence of unusually large and irregularly shaped boutons and with a marked reduction in the number of glutamatergic type Ib boutons (Figure 3A, 3B, and 3F). Close examination of the NMJs in these mutants revealed that there was also a dramatic increase in the amount of presynaptic debris (Figure 3C–3E, arrows, 3H) and number of ghost boutons (Figure 3E, arrowheads, 3G). Interestingly, we also found that third instar *draper* mutant larvae had reduced larval motility in behavioral assays (Figure S1), suggesting that the accumulation of presynaptically shed material may adversely affect neuromuscular function. Thus, in the absence of Draper function NMJs develop abnormally and presynaptic debris and ghost boutons accumulate at high levels. These observations suggest that an engulfing cell type might invade, or be a resident component of, the NMJ, and phagocytose shed presynaptic material.

### Draper Is Expressed in Muscle and Glia and Glial Cells Establish Transient Interactions with the NMJ

**Figure 4:**
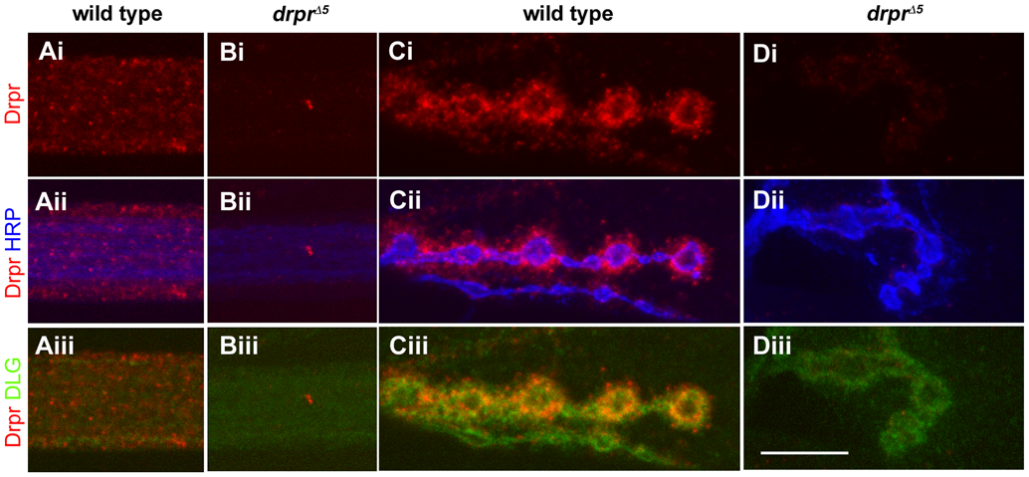
Draper is expressed in peripheral glia and in the postsynaptic region of the NMJ. Wild-type and *draper* ^Δ5^ null mutant third instar larvae were stained with α-Draper (red), α-HRP (blue), and α-DLG antibodies (green). (Ai–iii) Draper was readily detectable in peripheral glia, which surround the HRP^+^ axons. (Bi–iii) Draper immunoreactivity is absent from peripheral nerves in *draper* ^Δ*5*^ null animals, demonstrating the specificity of α-Draper sera for Draper in the segmental nerves. (Ci–iii) Draper is present postsynaptically at the NMJ surrounding HRP^+^ presynaptic boutons (Cii), and colocalizes with the primarily postsynaptic marker DLG (Ciii). (Di–iii) Draper immunoreactivity is absent from the NMJ in *draper* ^Δ*5*^ null animals. Calibration scale is 9.0 µm.

In the fly nervous system Draper is expressed in glia where it has crucial roles in engulfment activity [23]–[26]. To determine if Draper was also present in glial cells at the NMJ, we used α-Draper antibodies [24]. Surprisingly, in addition to its localization in peripheral glia that wrap around motor nerves (Figure 4A), we found that Draper immunoreactivity was present at the postsynaptic region of every synaptic bouton in colocalization with the *Drosophila* PSD-95 homolog DLG (Figure 4C). This immunoreactivity was specific to Draper, as it was virtually eliminated in *draper* null mutants (Figure 4B and 4D).

**Figure 5:**
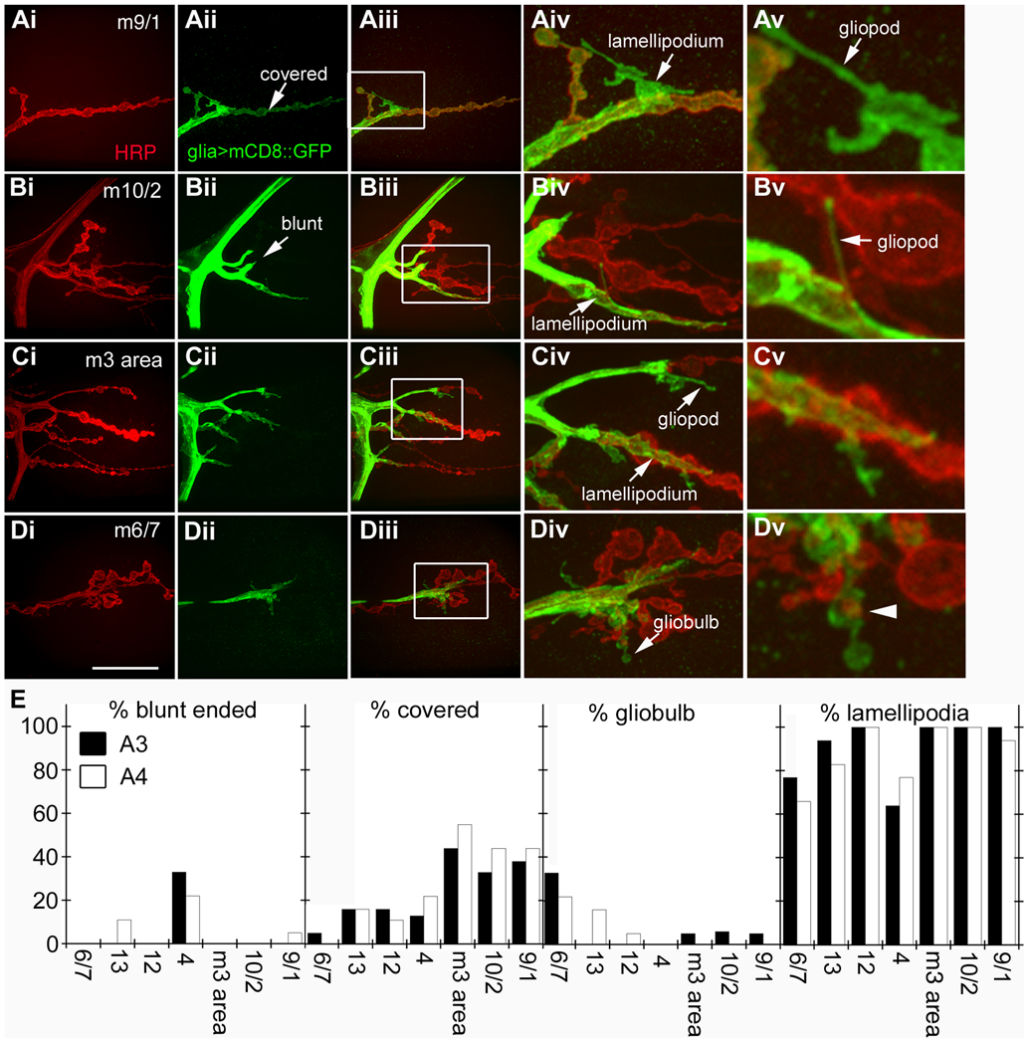
Glial cells dynamically invade the larval NMJ and their membrane extensions exhibit diverse morphologies. Glial processes at the NMJ were observed by expressing mCD8-GFP in glia (with the Gli-Gal4 driver) and staining with α-HRP (red) and α-GFP (green) antibodies. Low magnification views of specific NMJs (identity indicated by the numbers in the panels) are presented in columns (i–iii). Higher magnification views of the boxed regions in column (iii) are shown in columns (iv) and (v). (Ai–v) In some cases, glial cell processes appear to cover the entire NMJ arbor (covered; Aii, arrow). Glial cells could also be found extending lamellipodia-like extensions away from the parent arbor (lamellipodium; Aiv, arrow), or smaller filopodia-like projections (gliopods; Av, arrow). (Bi–v) In many cases glial cell processes terminated at the branch point where the motor axon entered the muscle field (blunt; Bii, arrow). When glial processes invaded the NMJ, gliopods could be found extending from one NMJ branch across to another (Bv, arrow). (Ci–v) An example of a gliopod extending into an area devoid of synaptic boutons (Civ, arrow), and the extension of a lamellipodium contacting several synaptic boutons as well as a muscle region devoid of boutons (Cv). (Di–v) Glial cellular extensions can take on a spherical shape similar to boutons (gliobulb; Div, arrow), which sometimes surrounds a synaptic bouton (Dv, arrowhead) or are devoid of synaptic boutons. (E) Quantification of glial projections at the third instar larval NMJ. The identity of muscles scored is indicated on the *x*-axis. “m3 area” (Ci, E) corresponds to NMJs at muscles 3, 19, 20, and 11. *n* = 20 hemisegments assayed. Calibration scale is 18 µm for (columns i–iii), 9 µm for (iv), and 4 µm for (v). *n* = 10 animals.

The above observation was surprising, since in contrast to vertebrate NMJs, where terminal Schwann cells completely cover the NMJ [27], at the glutamatergic *Drosophila* larval NMJ terminal glia have not been reported to cap the synaptic arbor [28],[29]. Instead, NMJ arbors are buried within the muscle surface, which wraps around the boutons forming a layered system of membranes, the subsynaptic reticulum (SSR) [30],[31]. Previous studies have suggested that at the larval NMJ peripheral glia ensheath the segmental nerve, but for the most part, their membranes terminate at the axon branch point or at the first synaptic bouton closest to the branch point [29]. The presence of Draper surrounding the entire NMJ led us to reexamine the organization of glial cell membranes at the NMJ and their relationship to synaptic boutons. For these experiments we expressed a membrane tethered GFP (mCD8-GFP) in peripheral glia, using Gliotactin-Gal4 (Gli-Gal4), and HRP-labeled NMJs from abdominal segments 3 and 4 were systematically examined in fixed preparations. We found that in the majority of cases glial membranes deeply invaded the NMJ (Figure 5), presumably invading the space between the presynaptic motorneuron terminal and the SSR. Some NMJs (2%–40% on average depending on the specific NMJ), particularly those innervating dorsal muscles, appeared completely covered by glial membranes (Figure 5A and 5E; covered NMJs). A majority (80%–100%) of NMJs were associated with lamellipodia-like glial extensions that contacted several boutons (Figure 5A–5C, and 5E). Glia also extended thin filopodia-like processes that contacted synaptic boutons at the same NMJ branch or that exited the branch and interacted with synaptic boutons from a different NMJ branch (Figure 5Av and 5Bv). Glial membrane processes were also observed in association with muscle regions around the NMJ that were completely devoid of synaptic boutons (Figure 5Aiv and 5Civ–v). A small percentage (∼7%) of glial extensions had an elliptical appearance and terminated in bulbous structures of variable size (Figure 5Div–v and 5E). These bulbous structures sometimes surrounded a synaptic bouton (Figure 5Dv, arrowhead). In some NMJs (11%–33%) glial membranes did not invade the NMJ and muscle, and terminated at the nerve branch-point before synaptic boutons (Figure 5Bi–iii; blunt ended).

Interestingly, the pattern of glial extensions was not stereotypic and showed a high degree of variability among segments and identified muscles from different individuals. This observation suggests that the glial processes are likely to extend and retract in a dynamic fashion. This possibility was examined by live imaging preparations expressing mCD8-GFP in peripheral glia with Gliotactin-Gal4. We found that glial processes were indeed at the NMJ, and extended or retracted within a period of minutes (Video S2). These observations indicate that glial cells at the larval NMJ have previously unappreciated dynamics, and that they establish multiple transient associations with the NMJ. However, our studies of Draper localization at the NMJ demonstrated that Draper is present at every NMJ and surrounding each synaptic bouton (Figure 4C). Thus, the extension of glial membranes is unlikely to account for Draper localization at the entire NMJ, raising the possibility that muscles might also contribute to NMJ Draper localization.

In *draper* mutants, there were some changes in the distribution and frequency of glial extensions. Glial extensions that covered the entire NMJ (covered NMJs) were absent or drastically reduced in frequency, and there were also changes in the distribution and frequency of gliobulbs (Figure S2). In contrast, there was a strong increase in the frequency of blunted projections (i.e., those that end close to the nerve branch point and do not interact with synaptic boutons), and a normal level of lamellipodia-like extensions). These observations suggest that in the absence of Draper function some glial membranes do not extend properly into the NMJ. Thus positive signaling through Draper, perhaps in response to cues released by presynaptic debris, may directly regulate a subset of glial membrane movements at the NMJ.

### Both Glia and Muscle Cells Act as Phagocytes and Clear Presynaptic Debris from the NMJ

**Figure 6:**
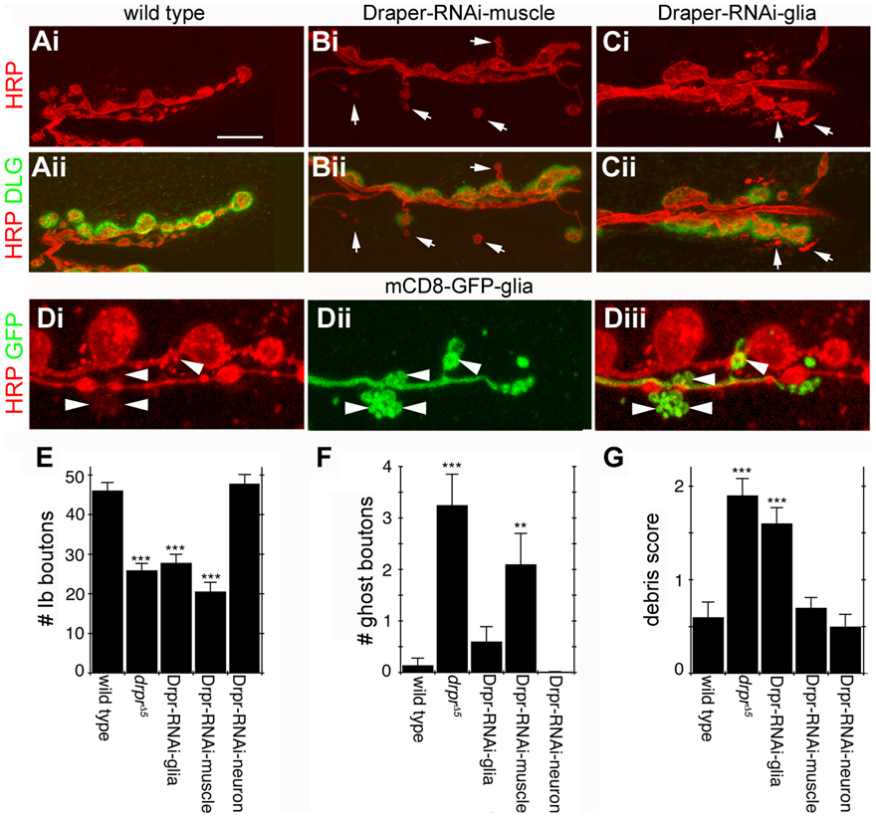
Draper function is essential in both glia and muscle cells for clearance of ghost boutons and shed presynaptic debris and for normal synaptic growth. Draper function was knocked-down by expressing UAS-Draper-RNAi in either muscle (C57-Gal4), glia (repo-Gal4), or motor neurons (OK6-Gal4), and ghost boutons and presynaptic debris were quantified by staining for HRP (red), and the postsynapse was visualized with DLG (green). (Ai–ii) Wild-type NMJs have very little presynaptic debris and few or no ghost boutons. (Bi–ii) Muscle-specific Draper knockdown leads to the accumulation of ghost boutons (arrows), but not of presynaptic debris. (Ci–ii) Glial-specific Draper knockdown leads to the accumulation of presynaptic debris (arrows), but not of ghost boutons. (D) mCD8-GFP (green) was expressed in glia with repo-Gal4 and motor neurons were visualized by staining for HRP (red). representative images of weak HRP signal detected within glial extensions (arrowheads). (E) Quantification of number of type Ib synaptic boutons at muscle 6/7 showing that Draper knockdown in glia or muscle cells reduces bouton number to those in *draper* ^Δ*5*^ null mutants, while Draper knockdown in motor neurons has no effect. (F) Quantification of ghost bouton number. Knockdown of Draper in muscle cells, but not glia or motor neurons, leads to the accumulation of ghost boutons at levels equivalent to those found in *draper* ^Δ*5*^ null mutants. (G) Quantification of shed presynaptic debris. Draper knockdown in glial cells, but not muscles or motorneurons, leads to the accumulation of presynaptic debris at levels similar to *draper* ^Δ*5*^ null mutants. ***, *p*<0.001; **, *p*≤0.01; *, *p*≤0.05. Calibration scale is 12 µm for (A and B), and 3 µm for (D). (*n*≥10 for each genotype).

To address the possibility that Draper might function both in glia and muscle to sculpt the NMJ we selectively expressed a Draper-RNAi designed to knockdown all Draper isoforms in glia or muscles using cell-specific Gal4 strains. RNAi knockdown of Draper in either muscle or glia resulted in a reduction in the number of synaptic boutons, which was not significantly different from the *draper* null mutant (Figure 6E). This indicates that the removal of Draper from either cell type is sufficient to interfere with NMJ growth. Remarkably, however, downregulating Draper in muscle versus glia had a different consequence for the deposition of presynaptic debris and the appearance of detached ghost boutons. RNAi knockdown of Draper in glia resulted in an increase in presynaptic debris to an extent similar to the *draper* null mutant (Figure 6C and 6G). However, no significant increase in the number of detached ghost boutons was observed (Figure 6F). If glial extensions are primarily involved in engulfing presynaptic debris, we predicted that we should find HRP positive debris within the glial extensions. We found that this was indeed the case. We found several instances in which glial terminals formed bulb-like structures that contained anti-HRP immunoreactive puncta within (Figure 6D, arrowheads).

In contrast, downregulating Draper in muscle resulted in an increase in the number of ghost boutons (Figure 6B and 6F), but the level of presynaptic debris was similar to wild type (Figure 6B and 6G). Expressing Draper RNAi in motorneurons did not affect the number of boutons, ghost boutons, or the levels of presynaptic debris (Figure 6E–6G). These results support the idea that Draper functions both in muscle and glia, and that the function of Draper in each cell has some degree of specialization. While glial Draper appears to function in removing presynaptic debris, muscle Draper appears to remove ghost boutons fated for elimination. Importantly, these observations also provide the first evidence that muscle cells fulfill a phagocytic function at the NMJ.

### Downregulation of Ced-6 Mimics Cell-Specific Draper Phenotypes at the NMJ

**Figure 7:**
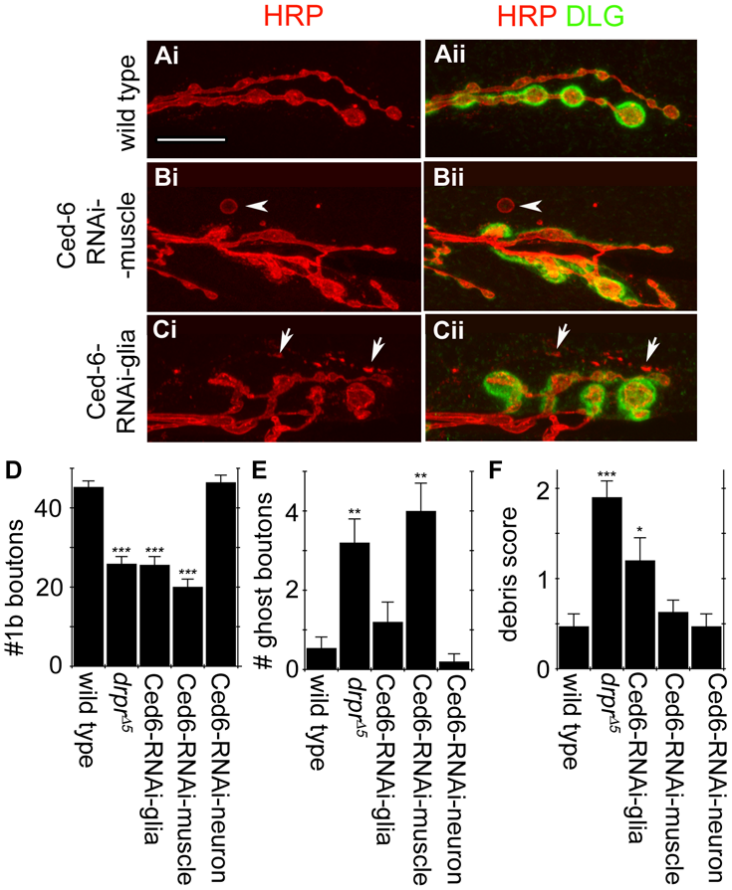
dCed-6, a key component of the Draper signaling pathway, is required for clearance of ghost boutons and presynaptic debris and for normal synaptic growth. dCed-6 function at the NMJ was assayed by expressing UAS-dCed-6-RNAi in glia, motor neurons, and muscles. Preparations were labeled with the presynaptic marker α-HRP (red) and the postsynaptic marker α-DLG (green). (Ai–ii) Wild-type NMJs exhibit little or no presynaptic debris and ghost boutons. (Bi–ii) Muscle-specific dCed-6 knockdown leads to the accumulation of ghost boutons (arrowheads) but very little presynaptic debris. (Ci–ii) Glial-specific dCed-6 knockdown leads to the accumulation of presynaptic debris (arrows) but not ghost boutons. (D–F) Quantification of the number of (D) type Ib boutons, (E) ghost boutons, and (F) presynaptic debris in control and dCed-6 knockdown backgrounds. dCed-6 function is required in both muscles and glia for (D) normal synaptic growth, in (E) muscles for the clearance of ghost boutons, and (F) in glia for clearance of presynaptic debris. ***, *p*<0.001; **, *p*≤0.01; *, *p*≤0.05. For (D–F), *n* = 12 for wild type, 9 for *drpr* ^Δ*5*^, and 13 for dCed-6^RNAi^. Calibration scale is 12 µm.

Previous studies have shown that the PTB-domain protein dCed-6 functions downstream of Draper [23]. Therefore, we used RNAi knockdown of dCed-6 in muscle or glia as a second approach to blocking glial and muscle engulfment activity. As in *draper* mutants, downregulating dCed-6 in either muscle or peripheral glia resulted in significant decrease in the number of synaptic boutons (Figure 7A–7D). In contrast, no effect was observed when dCed-6-RNAi was expressed in motorneurons (Figure 7D). Similar to Draper RNAi knockdown, expressing dCed-6-RNAi in muscles or glia had differential consequences for the appearance of presynaptic debris versus ghost boutons. Decreased levels of dCed-6 in muscles led to an increase in the number of ghost boutons, but had no influence in the deposition of presynaptic debris (Figure 7B, 7E, and 7F). Downregulating dCed-6 in glia, on the other hand, led to a significant increase in presynaptic debris deposition, but the number of ghost boutons remained unaltered (Figure 7C, 7E, and 7F). These results are consistent with the notion that dCed-6 functions downstream of Draper during the development of the NMJ. Further, they support the model that both muscle and glia contribute differentially to the clearance of debris versus ghost boutons at the NMJ.

### Accumulation of Presynaptic Debris, Ghost Boutons, and Defects in NMJ Growth Map to the *draper* Gene

**Figure 8:**
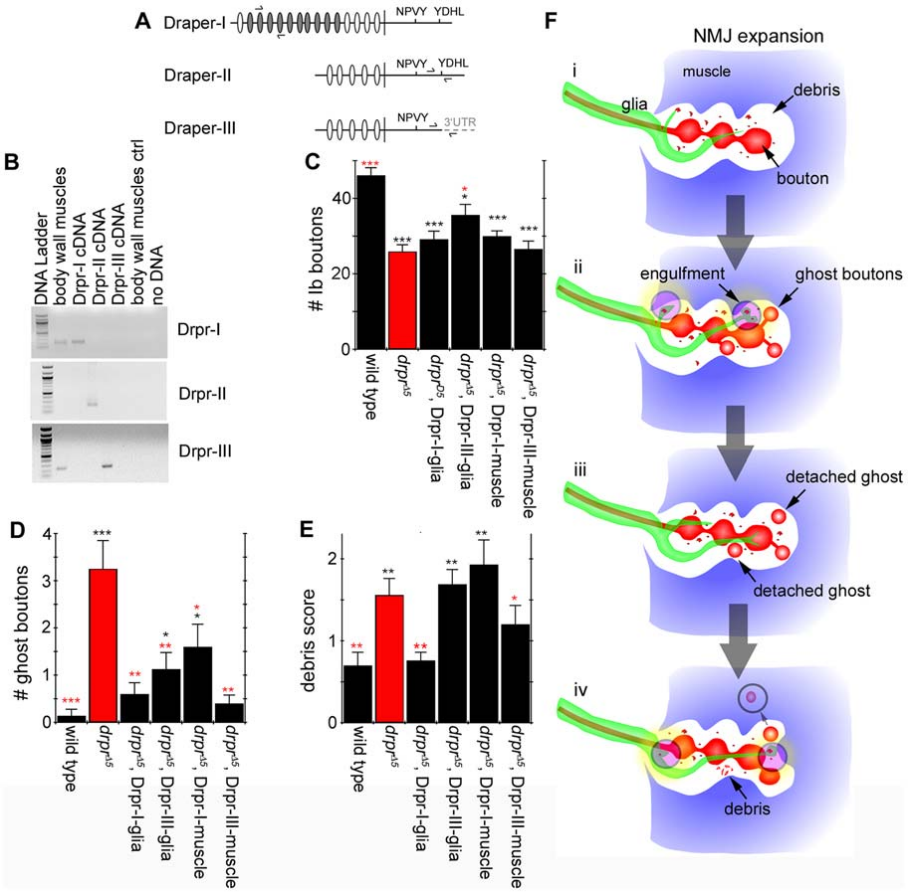
Cell type-specific rescue of *draper* mutant phenotypes with alternative Draper receptor isoforms. (A) Three isoforms of the Draper receptor have been identified in *Drosophila* [24]. We designed isoform-specific primers (arrows) to determine the presence of each unique isoform in larvae. Ovals represent EGF-like repeats in the extracellular domain. (B) RT-PCR shows that Draper-I and Draper-III are expressed in body wall muscles. cDNAs for each isoform were used as positive controls, along with a minus RT reaction. (C–E) To assay for the cell-specific function of Draper-I or Draper-III, each isoform was expressed in either glia (with Gli-Gal4) or muscle cells (with C57-Gal4) in *draper* ^Δ*5*^ null mutant backgrounds to determine which isoform rescued mutant phenotypes, including (C) decreased bouton number, (D) accumulation of ghost boutons, and (E) accumulation of presynaptic debris. *draper* ^Δ*5*^ mutant phenotypes are shown in red bars. (C) Expression of Draper-III in glia provides a partial rescue of the decrease in type Ib bouton number observed in *draper* ^Δ*5*^ mutants. (D) Expression of Draper-I in glia or Draper-III in muscle or glia provides complete rescue of the accumulation of ghost boutons observed in *draper* ^Δ*5*^ mutants. Expression of Draper-III in glia or Draper-I in muscle also provides a partial rescue of ghost bouton number. (E) Expression of Draper-I in glia fully rescues the accumulation of presynaptic debris observed in *draper* ^Δ*5*^ mutants. Expression of Draper-III in muscle also provides weak but significant rescue. (F) Model for Draper receptor function at the NMJ. (i) A motorneuron with an increase in activity or other developmental cues produces (ii) more ghost boutons, and an increase in debris that is engulfed by glial extensions. The newly formed ghost boutons will either (iii) stabilize or detach from the main arbor. Detached boutons will either (iv) degrade into debris or be engulfed by the muscle. For (C–E), ***, *p*<0.001; **, *p*≤0.01; *, *p*≤0.05. Red asterisk, compared to *draper* ^Δ*5*^ mutants; black asterisk, compared to wild type. For (C–E), *n* = 9, 9, 8, 8, 8, 8, for genotype as listed left→right, respectively.

The *draper* gene gives rise to three different Draper isoforms, each with a unique combination of intracellular and extracellular domains (Figure 8A). Draper-I bears 15 extracellular EGF repeats, whereas Draper-II and -III only contain five [24]. In their intracellular domains, all isoforms contain a potential dCed-6 binding site (NPXY), but the Shark binding site is only present in Draper-I and -II. To determine which of the isoforms might be involved in NMJ development, we first carried out reverse-transcription PCR (RT-PCR) of body wall muscles. Interestingly, we found that Draper-I and III, but not Draper-II were expressed at the neuromuscular system (Figure 8A and 8B). Therefore, we carried out rescue experiments by expressing Draper-I or -III in muscles or glia in a *draper* null mutant background.

None of the Draper isoforms completely rescued the decrease in bouton number observed in the *drpr* null (Figure 8C). This is consistent with the observations with cell-specific Draper-RNAi expression, showing that Draper functions both in muscle and glia, and that downregulating Draper in either cell is sufficient to decrease bouton number to an extent similar to the *draper* null mutant alone. In the case of ghost boutons, expressing Draper-I in glia or Draper-III in muscle completely rescued the mutant phenotype (Figure 8D). However, expressing Draper III in glia or Draper I in muscle also resulted in substantial but incomplete rescue. For the deposition of presynaptic debris, only expressing Drpr-I in glia completely rescued the phenotype, but partial rescue was also observed when Drpr-III was expressed in muscle (Figure 8E). These data provide conclusive evidence that the phenotypes we observe in *draper* null mutant NMJs indeed map to the *draper* gene, and that the phenotypes we observe in *draper* mutants can be significantly rescued by resupplying Draper in glia or muscle cells (Figure 8F). The incomplete rescue of some of the phenotypes by specific isoforms might represent redundant functions by these isoforms, a requirement for multiple isoforms for complete rescue, or simply result from increased Draper expression in transgenic animals.

## Discussion

Here we have studied the in vivo dynamics of synaptic connectivity between single motor inputs and their target muscle cells. We describe a novel event that occurs during the remodeling of single synaptic arbors during development or activity-induced plasticity: the shedding of presynaptic debris and aborted synaptic boutons that failed to stabilize. This process differs from developmental pruning or intercellular competition during synapse elimination, as in those cases entire nerve terminals are eliminated, thereby changing the wiring diagram of a circuit. Rather, we show that the expansion of an already established synaptic input involves significant production of presynaptic membrane debris and the detachment of undifferentiated synaptic boutons destined for elimination from the main arbor. Both glial and muscle cells act in concert to clear the developing NMJ of this shed presynaptic material, and the suppression of engulfing activity in glial or muscle cells leads to highly disrupted NMJ growth. We propose that this novel mechanism might serve to rapidly adapt the size of a growing synaptic terminal to the changing demands of the target cell by shifting the equilibrium between synapse stabilization and synapse destabilization.

### Expanding Presynaptic Arbors Shed Membrane Debris in an Activity-Dependent Manner

During larval development, the NMJ is continuously increasing the size and number of synaptic boutons. This expansion serves as a compensatory mechanism to preserve synaptic strength, despite the massive growth of muscle cells [32]. Our studies provide evidence that normal NMJ growth includes the constitutive shedding of presynaptic membranes. The presynaptic origin of HRP-positive debris was demonstrated by labeling motorneuron membranes with genetically encoded mCD8-GFP, which consistently labeled the debris, by the observation that in some cases ghost boutons that detached from the main arbor disintegrated into debris, and by the finding that the debris also contained presynaptic proteins, such as CSP. Thus, synaptic debris might contain synaptic vesicles or vesicle membrane remnants that failed to be recycled. Interestingly, Brp, an active zone marker [33], was absent from the debris. This absence might reflect its degradation, or alternatively, the derivation of presynaptic debris from periactive regions of the NMJ. Indeed, FasII, which is localized at periactive zones [34] was also present in presynaptic debris.

Acute spaced stimulation of the larval NMJ leads to the formation of dynamically extending and retracting synaptopods, and to the appearance of ghost boutons [13]. While some ghost boutons differentiate by acquiring active zones and postsynaptic proteins [13], here we found that others lost their connection with the presynaptic arbor and were specifically removed. What happens to ghost boutons that detach from the main arbor? In most cases we found that detached ghost boutons rapidly disappeared from the NMJ. On the basis of our finding that suppressing engulfing action in muscle leads to the accumulation of ghost boutons, we propose that these are engulfed directly by muscle cells (Figure 8F).

In other cases we found that ghost boutons, along with the stalk by which they were initially attached to the main arbor, would degenerate into smaller fragments resembling presynaptic debris. Thus at some level, ghost boutons also appear to be able to disintegrate into presynaptic debris. That presynaptic debris and ghost boutons are unique cellular remnants is also argued by the fact that they are differentially engulfed by glia and muscle cells, respectively (Figure 8F). Nevertheless, the detachment and elimination of ghost boutons we describe represents a simple and newly defined mechanism for the removal of excessive synapses formed by individual innervating motorneurons. This process might also serve as a mechanism for rapid stabilization of new synaptic boutons during, for example, periods of increased synaptic or locomotor activity (see below) [13],[35],[36].

The functional significance of shedding presynaptic debris remains unclear. Manipulations that promote rapid synaptic growth, such as acute spaced stimulation, lead to an increase in presynaptic debris suggesting that its production is associated with synaptic growth. While some presynaptic debris appears to be derived from the breakdown of disconnected ghost boutons, we also observed the *de novo* formation of presynaptic debris in the absence of any ghost boutons. Thus, presynaptic debris is likely directly shed by motorneuron endings. Presynaptically shed debris might derive from dynamically extending synaptopods, whose formation is dramatically enhanced by increasing neural activity [13]. However, in live preparations demonstrating robust synaptopod growth we have yet to directly observe the formation of debris following synaptopod expansion or retraction (Gorczyca M, Ashley J, Fuentes-Medel Y, unpublished data).

The presence of presynaptic debris might highlight the extremely dynamic nature of synapse addition in vivo. Two important mechanisms appear to operate during NMJ expansion. First, the NMJ is shaped by a homeostatic mechanism that maintains synaptic efficacy despite larval muscle growth [32]. Second, the NMJ has the ability to respond to acute changes in activity and sensory experience with rapid modifications in synaptic structure and function. Well-fed larvae placed in a substrate devoid of food show an increase in synaptic strength within 30 min [35], and spaced stimulation induces robust synaptic growth within 2 h [13]. It is tempting to speculate that presynaptic shedding is the byproduct of a mechanism designed to ensure rapid and efficient changes in synaptic performance. For example, the initiation of synaptic bouton formation might be a continuous process. This pool of synaptic boutons might reach an immature stage and if not subsequently stabilized by activity or other signals they might be shed and removed. Such a mechanism would provide a continuous supply of immature boutons ready to stabilize if rapid growth becomes essential.

### Synaptic Debris and Ghost Boutons Are Engulfed by Glial and Muscle Cells

Glial cells have a key role in the removal of axonal debris and neuronal cell corpses from the central nervous system [22],[37], but mounting evidence also implicates glial cells in the elimination of synaptic inputs. In mammals microglia rapidly spread along neurites of injured motorneurons and displace synaptic inputs through synaptic stripping [38]. At the mammalian NMJ, terminal Schwann cells are also active participants in the activity-dependent elimination of exuberant motorneuron inputs by apparently pinching off fragments of retracting terminals [11].

Here we describe a novel mechanism by which glia, through their phagocytic clearance of shed synaptic debris, can sculpt synaptic connectivity within a single arbor and ultimately modulate the growth of nerve terminals. The formation of shed presynaptic material appears to be autonomous and not require the engulfing action of glial cells since presynaptic debris and ghost boutons accumulate at high levels in *draper* mutants. Notably, muscle cells collaborated with glia in the removal of shed presynaptic membranes and thus also helped to sculpt the growing NMJ. These observations provide a new view on the role of muscle cells in regulating synaptic growth: muscle cells are not simply postsynaptic target cells that give and receive synaptogenic signals; they are also phagocytes at the NMJ and through engulfing shed presynaptic material can help shape synaptic connectivity.

Why has such presynaptic material not been previously described at the well-studied *Drosophila* NMJ? This is likely due to the fact that we have assayed NMJ morphology for the first time in engulfment mutants. Even in wild type a very low level of presynaptic debris (this report) and a small number of ghost boutons [13] is observed. However in *draper* mutants or knockdown animals we observe their dramatic accumulation, which is reminiscent of the process of cell corpse engulfment after apoptotic cell death. Cell corpses are rapidly engulfed during development and thus very few are observed in wild-type animals. In contrast, they accumulate at significant levels in animals with reduced cell corpse engulfment activity, such as *C. elegans ced-1* or *ced-6* mutants [39].

We found that glial cells extended membrane processes that deeply invaded the NMJ. These cellular interactions were highly dynamic, as demonstrated by our time-lapse imaging, and by the high variability in the extent and type of glial membrane projections we found at the NMJ. Some projections were in the form of thin gliopods that associated with boutons within a branch or that extended across branches. Others resembled flat lamellipodia that associated with synaptic boutons or with the muscle. Given the requirement for glial Draper in the removal of synaptic debris, it is tempting to speculate that glial membranes are continuously and dynamically surveying the NMJ for the presence of synaptic debris, which is then engulfed. Consistent with this notion, we found several examples of glial membranes extending away from the arbor and overlapping with presynaptic debris. We also found that in some cases, HRP positive fragments were found associated with bulbous structures formed by the glial projections, suggesting that glia can engulf presynaptic debris. We also observed glial membrane projections that had the form of boutons, sometimes draping over an entire bouton, or extending well beyond the terminal bouton. While the function of these structures remains unclear we envisage at least two potential roles. First, these might represent glial extensions actively engulfing ghost boutons, although this would be predicted to be a rare event since our cell-type specific analyses argue that muscle cells are primarily responsible for clearance of ghost boutons. Second, these extensions, along with the additional types described above that extend beyond axonal arbors into the muscle, could be physically opening up space in the muscle cell for new bouton formation or process extension.

### Recognition and Clearance of Shed Presynaptic Debris and Ghost Boutons Requires the Draper Signaling Pathway

Interestingly, we found that in *draper* mutants both disconnected ghost boutons and presynaptic debris accumulated, and this accumulation had a negative effect on NMJ expansion and bouton morphology. Moreover, synaptic growth appeared to be highly sensitive to both types of shed presynaptic material since the accumulation of either ghost boutons or presynaptic debris (when engulfment activity was blocked in muscles or glia, respectively) led to reductions in bouton growth similar to that seen in *draper* null mutants. As mentioned above, shed material might contain important signaling factors that potently stimulate or inhibit new synapse formation. If, for example, presynaptic debris contains molecules that inhibit synaptogenesis, the accumulation of such material would be expected to negatively regulate synaptic growth. Perhaps a similar type of inappropriate modulation of synaptogenesis by the membrane fragments of pruned terminals also accounts for their rapid clearance from the central nervous system after degeneration.

*Drosophila* glial cells also engulf neuronal cell corpses and pruned or degenerating axons. Each of these targets is generated by a unique degenerative molecular cascade: cell corpses are produced by canonical apoptotic cell death pathways [40], pruned axons undergo degeneration through a ubiquitin proteasome-dependent mechanism [41], and severed axons undergo Wallerian degeneration via Wld^s^-modulated mechanisms [26]. Despite their unique pathways of production, each is engulfed by glia through Draper-dependent mechanisms, implying that these engulfment targets autonomously tag themselves with molecularly similar “eat me” cues. Our observations that mutations in *draper* led to accumulation of presynaptic debris and detached ghost boutons suggests that these new glial/muscle engulfment targets also produce similar cues for phagocytic cells to promote their destruction. If so, these data argue that all the necessary machinery essential for tagging membrane fragments for engulfment are present in a ghost bouton or fragment of presynaptic membrane. Importantly, while a lack of glial-mediated clearance of several targets has been observed in vivo—cell corpses, pruned axons or dendrites, and axons undergoing Wallerian degeneration—almost nothing is known about phenotypic consequences of a lack of glial engulfment function in the nervous system. Here we demonstrate that failure of glia and muscle to clear presynaptically derived material negatively regulates synaptic growth.

In conclusion our studies demonstrate that the process of synaptic growth includes a significant degree of membrane/synaptic instability, and that growing terminals are constantly sloughing off undifferentiated boutons and fragments of membrane. Our observations demonstrate that growing NMJs generate an excess number of undifferentiated synaptic boutons and that only a fraction becomes stabilized and drive the assembly of the postsynaptic apparatus. Exuberant synapses that have failed to form successful postsynaptic contacts are shed, and cleared from the NMJ by glia and muscle cells. The presence of such a pool ensures a continuous supply of nascent synapses available for use to rapidly increase input into the muscle if dictated by dynamic changes in signaling at the NMJ.

## Materials and Methods

### *Drosophila* Strains and Behavioral Assays

The following fly strains were used for this study: *draper* ^Δ*5*^ and UAS-Draper-RNAi [26], UAS-dCed-6-RNAi [23]; Repo-Gal4 (a gift from B. Jones), Gli-Gal4 [42], OK6-Gal4 [16], C57-Gal4 and C380-Gal4 [20], UAS-mCD8-GFP [43] UAS-myrRFP (Bloomington Stock Center), MHC-mCD8GFP-Sh [21], and UAS-ChR2 [44]. UAS-Draper-I and UAS-Draper-III were generated by M.A. Logan and will be described in detail elsewhere (MAL and MRF, unpublished data). For larval motility assays, larvae were cultured at 25°C, wandering third instar larvae were collected, briefly washed in distilled water, transferred to the center of a square agar plate, and covered with a transparent lid. After 30 s, total larval movement was followed for 1 min under red light conditions, 60% humidity, at 25°C.

### Immunolabeling, Live-Imaging, and Confocal Microscopy

Third instar *Drosophila* larvae were dissected in calcium free saline [45] and fixed for 10 min with nonalcoholic Bouin's solution unless otherwise noted. Primary antibodies were used at the following dilutions: α-Draper, 1∶5,000 [24]; rabbit α-DLG, 1∶20,000 [46]; mouse α-DLG, 1∶500 (clone 4F3, Developmental Studies Hybridoma Bank, DSHB); α-CSP, 1∶100 [47]; α-Synapsin, 1∶10 (a gift from E. Buchner; [48]; α-Fas II, 1∶3000 [46]; α-GFP, 1∶200 (Molecular Probes); nc82 (α-Brp), 1∶100 (DSHB); FITC or Texas red-conjugated α-HRP 1∶200 (Jackson Immunoresearch). Secondary antibodies conjugated to FITC, Texas Red, or Cy5 (Jackson Immunoresearch) were used at a concentration of 1∶200. Samples were imaged using a Zeiss Pascal confocal microscope and analyzed using the Zeiss LSM software package and ImageJ.

To study the organization of glial membranes at the NMJ we fixed larval body wall muscle preparations of controls and *draper* mutants expressing mCD8-GFP in glia using the Gli-Gal4 strain for 15 min in 4% paraformaldehyde fix, and double stained the preparations with Texas Red conjugated α-HRP 1∶200 (Jackson Immunoresearch) and α-GFP (Molecular Probes). Glial membrane extensions at identified body wall muscle NMJs from abdominal segments A3 and A4 were scored individually as “blunt ended” (glial membranes terminated at the branch point), “covered” (glial membranes completely ensheathed the NMJ), “gliobulbs” (glial extensions terminated in a bulbous structure), “gliopods” (small finger-like glial membrane projections), and lamellipodia (glial membranes formed flat extensions that partially covered the NMJ). The percentage of NMJs containing the above types of glial membranes projections was calculated from 20 hemisegments for controls, and 15 hemisegments for *draper* ^Δ*5*^ mutants.

Presynaptic debris was scored from type Ib boutons at muscles 6 and 7, abdominal segment A3. This quantification was performed using images of α-HRP labeled NMJs that were acquired with identical confocal settings, and the amount of debris scored blindly according to a subjective scale of 0–3. Number of NMJs analyzed are ten to 12 per sample (from six animals). To score presynaptic debris after spaced stimulation, intact larvae expressing channelrhodopsin-2 in motorneurons were subjected to spaced light stimulation as in (Ataman et al. [13]), fixed at 2 h (1.5 h stimulation, 30 min rest) (*n* = 18 for stimulated samples, *n* = 12 for unstimulated controls), and 18 h after stimulation (*n* = 6 for stimulated samples, *n* = 6 for unstimulated controls), and stained with α-HRP antibodies. Confocal images of NMJs at muscles 6 and 7 (A2 and A3) were acquired with identical settings, and two 8-µm diameter circles at the postsynaptic region of each NMJ branch were selected for analysis using NIH Image software. The number of synaptic boutons and ghost boutons were quantified at muscles 6 and 7 (A3) from preparations double stained with α-HRP and α-DLG (*n*≥10 NMJs per genotype). Data were represented in histograms as the average±SEM. Statistical significance of the data was obtained in pair-wise comparisons using the Student's *t*-test.

Live imagining of larvae was performed on either intact or dissected preps as Ataman et al. [13]. Briefly intact larvae were anesthetized using Sevoflurane (Baxter) and the dorsal muscles were then imaged through the cuticle using a 40× 1.2 NA objective on an Improvision spinning disk confocal microscope. Larvae were examined live by expression of UAS-mCD8GFP in motor neurons (pre-Gal4) or glia (gli-Gal4). Increased activity was induced in these larvae by expression of UAS-Channelrhodopsin2, and exposure to a pulsed 491-nm LED paradigm described in Ataman et al. [13] and Figure 1H. Larvae were examined every hour, every 4 h, or at 18-h intervals depending on the experiment. In order to visualize the debris, samples were converted to rainbow gradient color, and then contrast enhanced until the main arbor was saturated, as the debris is much dimmer than the presynaptic membrane.

Live imaging of glia was also performed in dissected preps, as Ataman et al. [13]. Briefly, larvae were dissected in 0.1 mM calcium *Drosophila* HL3-saline, and imaged on a Zeiss Pascal Confocal (Carl Zeiss) using either 25× or 40× water immersion objectives.

### RT-PCR

Total RNA was isolated from third instar body wall muscle preparations with Trizol (Invitrogen) and purified using the RNeasy Mini Kit (QIAGEN). First strand cDNA was synthesized using Superscript II (Invitrogen) enzyme and oligo (dT) 12–18 primer (Invitrogen). PCR was performed using the following Draper isoform specific primers to detect expression of Draper-I, Draper-II, or Draper-III: DrprIuECDF (5′-GGGTCCCCTATGTGATATGC-3′) and DrprIuECDR (5′-TTGTAGCACTCGCAGCTCTC-3′); DrprIIuF (5′-GAAAATATATAGCAAGATTTTGTTTCC-3′) and DrprIIuR (5′-TTCGTGTTGTCGAAGCACTC-3′); DrprIIIuF (5′-GTCATTAGACTTTTACACAGG c-3′) and DrprIIIuR (5′-CTAGCGTATAGAATCAGAC-3′). Plasmids containing the Draper isoforms (pUAST-DraperI, pUAST-DraperII, and pUAST-DraperIII) were used as controls for PCR amplification. PCR program was as follows: denature at 95°C for 1 min, anneal at 56°C for 30 s, extension at 72°C for 30 s (30 cycles total). PCR products were run on a 0.8% agarose gel and visualized by ethidium bromide stain.

## Supporting Information

Figure S1 ***draper*** **mutants exhibit reduced larval motility.** Wild-type controls (*CS* and *w^1118^*) were compared to *draper* ^Δ*5*^ mutant larvae in larval crawling assays (see Methods). *draper* mutants show reduced rates of locomotion (*p*<0.001).
(4.30 MB TIF)

Figure S2 **Changes in glial membrane extensions in** ***draper*** **mutants.** Glial membrane extensions in *draper* ^Δ*5*^ mutants were compared to controls by labeling membranes with mCD8-GFP (see Figure 5 and Methods). A3 and A4 correspond to abdominal segments. The identity of muscles scored is indicated on the *x*-axis. “m3 area” corresponds to NMJs at muscles 3, 19, 20, and 11. *n* = 15 hemisegments. *draper* ^Δ*5*^ mutants showed a dramatic decrease in the number of covered NMJs, a change in the distribution of gliobulbs, and an increase in the number of blunt ended glial projections.
(13.17 MB TIF)

Video S1 **Active disintegration of ghost boutons into smaller structures and disappearance from the NMJ.** Motorneurons were labeled with mCD8-GFP (using *C380-Gal4*), and imaged every 10 s for a 5-min interval. Note that one ghost bouton (center of field of view) splits into two smaller GFP^+^ structures, one lingers at the NMJ, while the other shifts its position dramatically and then disappears from the plane of focus. Full analysis of the Z-stack revealed that this particle had moved to a position deep within the muscle cell (unpublished data), apparently having been engulfed. A 3-D rendering of the Z-stack revealed that the presynaptic debris particles imaged remained fully within the Z-series and the changes observed were not the result of specimen drift.
(0.88 MB AVI)

Video S2 **Glial cells rapidly invade the NMJ in vivo.** Peripheral glia were labeled with mCD8-GFP (using the Gli-Gal4 driver), and glial dynamics at the NMJ were assayed in living third instar larvae. Total video length is 6 min. Note the extension of gliopods at the distal tip, and spreading of glial membranes at the branch point.
(4.56 MB AVI)

## Abbreviations

Brp: Bruchpilot
ChR2: channelrhodopsin-2
CSP: Cysteine string protein
DLG: Discs-Large
Fas: Fasciclin
GFP: green fluorescent protein
MHC: myosin heavy chain
NMJ: neuromuscular junction
Syn: Synapsin
