# Supplementary figures and images for: Glia and Muscle Sculpt Neuromuscular Arbors by Engulfing Destabilized Synaptic Boutons and Shed Presynaptic Debris
Source: PLoS Biol. 2009 Aug 25;7(8):e1000184. doi: 10.1371/journal.pbio.1000184 (PMC2724735; doi:10.1371/journal.pbio.1000184)

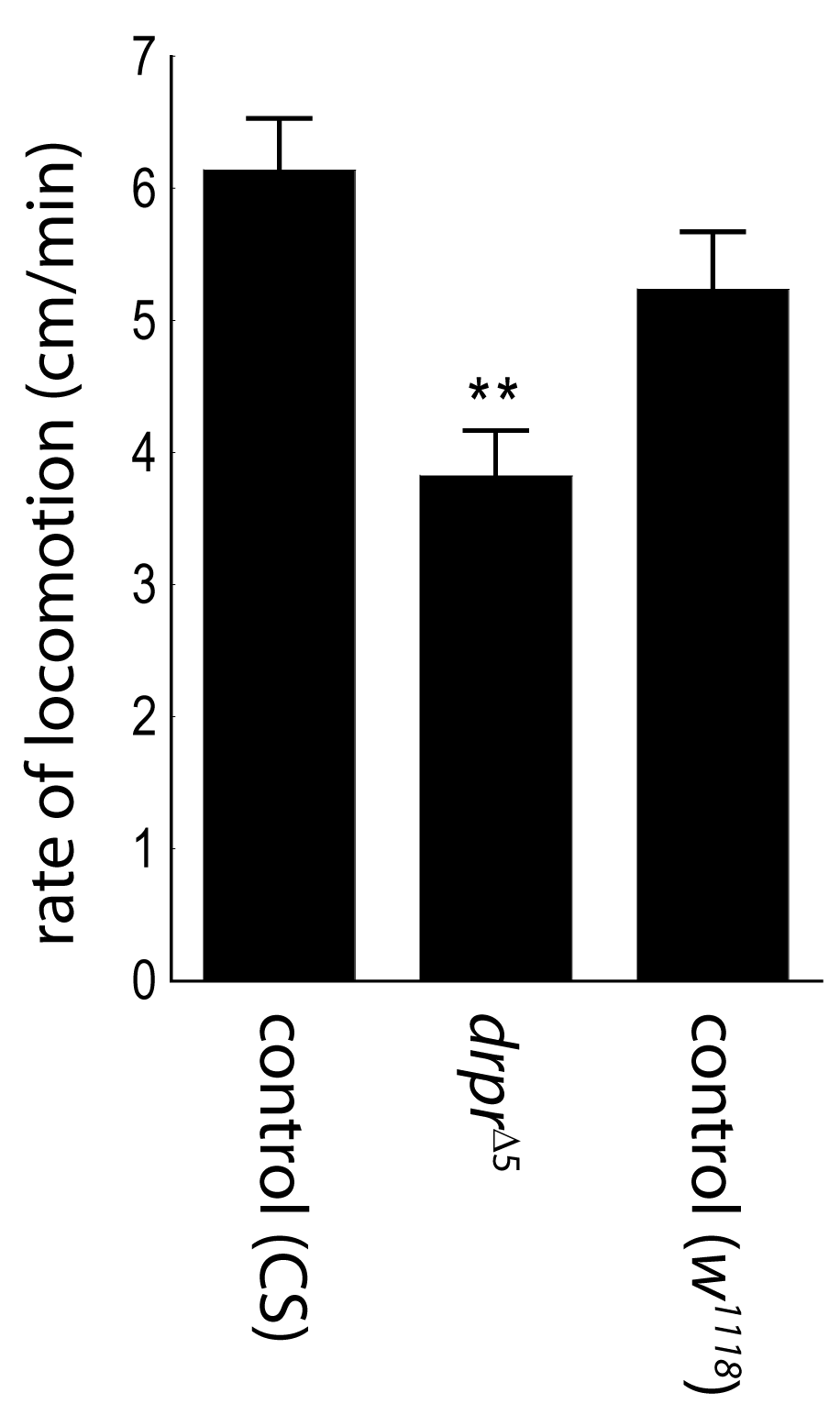

Supplement: Figure S1 — draper mutants exhibit reduced larval motility. Wild-type controls (CS and w1118) were compared to draper Δ5 mutant larvae in larval crawling assays (see Methods). draper mutants show reduced rates of locomotion (p<0.001). (4.30 MB TIF) [file pbio.1000184.s001.tif]

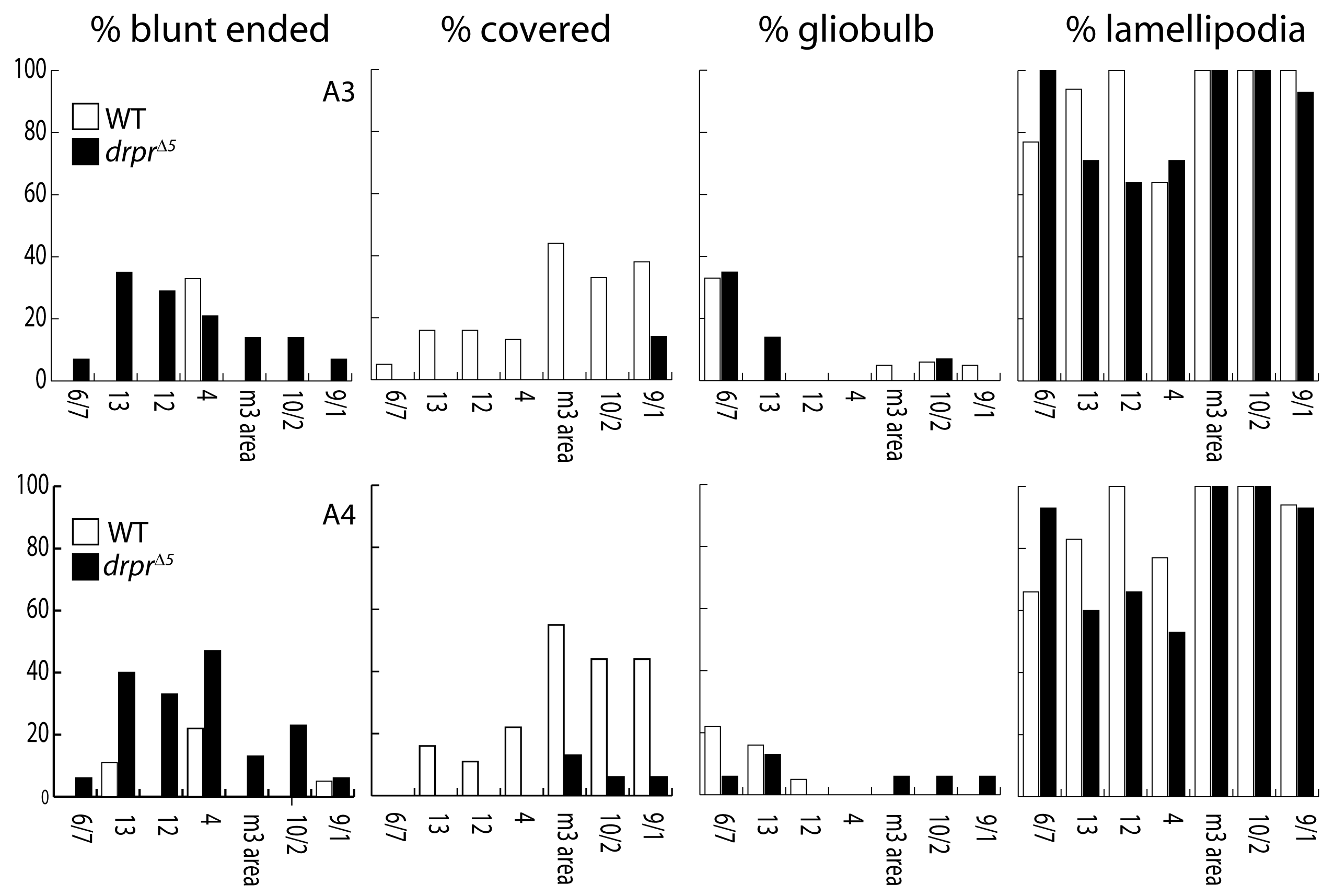

Supplement: Figure S2 — Changes in glial membrane extensions in draper mutants. Glial membrane extensions in draper Δ5 mutants were compared to controls by labeling membranes with mCD8-GFP (see Figure 5 and Methods). A3 and A4 correspond to abdominal segments. The identity of muscles scored is indicated on the x-axis. “m3 area” corresponds to NMJs at muscles 3, 19, 20, and 11. n = 15 hemisegments. draper Δ5 mutants showed a dramatic decrease in the number of covered NMJs, a change in the distribution of gliobulbs, and an increase in the number of blunt ended glial projections. (13.17 MB TIF) [file pbio.1000184.s002.tif]
